# T1 mapping allows the study of the development of oedema in a small animal model of Ischemia-Reperfusion

**DOI:** 10.1186/1532-429X-16-S1-P16

**Published:** 2014-01-16

**Authors:** Darach O h-Ici, Sarah Jeuthe, Felix Berger, Thore Dietrich, Titus Kuehne, Sebastian Kozerke, Daniel Messroghli

**Affiliations:** 1 Cardiovascular Imaging, Deutsches Herzzentrum Berlin, Berlin, Germany; 2 Division of Imaging Sciences & Biomedical Engineering, King's College London, London, UK; 3 Institute for Biomedical Engineering, ETH and University of Zurich, Zurich, Switzerland

## Background

Ischemic cell death is characterised by cellular oedema. Reperfusion may be expected to increase myocardial oedema via hyperaemia and osmotic changes. Cardiovascular magnetic resonance (CMR) can be used to detect oedema using T2 weighted imaging, but this technique does not allow the study of possible changes in oedema with reperfusion. The purpose of this study was to examine the development of oedema in a small animal model of Ischemia-Reperfusion using serial T1 mapping.

## Methods

Rats (n = 8) underwent coronary occlusion for 30 minutes followed by 60 minutes of reperfusion to delineate the time course of development of changes in non-contrast T1 abnormalities. T1 was quantified by 3.0 T CMR (Phillips) using a Small Animal Look-Locker Inversion Recovery (SALLI) sequence. T1 was quantified over time starting from baseline prior to occlusion in the remote zone and in the area at risk. Following the experiment the hearts were removed. The coronary artery was re-occluded to allow delineation of the area at risk with evans blue, then stained using triphenyltetrazolium chloride (TTC). This defined 1-Area at Risk, 2-Infarction [white], 3-Salvaged myocardium [stained red from TTC], and 4-Remote [Stained blue].

## Results

On coronary occlusion all rats developed myocardial ischemia initially confirmed by ECG changes and ventricular arrhythmia. Fatal arrhythmia occurred in 2 Rats. During occlusion T1 increased in the area at risk (baseline 1103 ± 79 increase to 1276 ± 64, p < 0.001). This increase was noted within the first ten minutes of coronary occlusion and remained unchanged during the 30 minute period of ischemia (p = 0.74) and following reperfusion at 30 (p = 0.83) and 60 minutes (p = 0.81).

## Conclusions

During coronary occlusion in rats, T1 increases in the area at risk consistent with the formation of ischemia. This change occurs with 10 minutes. Following reperfusion after a brief period of ischemia, T1 remains unchanged, indicating that reperfusion does not alter the degree of myocardial oedema in the the first hour after reperfusion.

## Funding

Darach O h-Ici is funded by a Sachmittelbeihilfe of the Deutsche Forschungsgemeinschaft (DFG) granted to Daniel Messroghli.

